# Raising the Alarmone: Within-Host Evolution of Antibiotic-Tolerant *Enterococcus faecium*

**DOI:** 10.1128/mBio.00066-17

**Published:** 2017-02-21

**Authors:** Daria Van Tyne, Michael S. Gilmore

**Affiliations:** a Department of Ophthalmology, Harvard Medical School, Massachusetts Eye and Ear Infirmary, Boston, Massachusetts, USA; b Department of Microbiology and Immunobiology, Harvard Medical School, Boston, Massachusetts, USA

## Abstract

Enterococci are ancient commensal bacteria that recently emerged as leading causes of antibiotic-resistant, hospital-acquired infection. Vancomycin-resistant enterococci (VRE) epitomize why drug-resistant enterococcal infections are a problem: VRE readily colonize the antibiotic-perturbed gastrointestinal (GI) tract where they amplify to large numbers, and from there, they infect other body sites, including the bloodstream, urinary tract, and surgical wounds. VRE are resistant to many antimicrobials and host defenses, which facilitates establishment at the site of infection and confounds therapeutic clearance. Having evolved to colonize the GI tract, VRE are comparatively ill adapted to the human bloodstream. A recent study by Honsa and colleagues (E. S. Honsa et al., mBio 8:e02124-16, 2017, https://doi.org/10.1128/mBio.02124-16) found that a strain of vancomycin-resistant *Enterococcus faecium* evolved antibiotic tolerance within the bloodstream of an immunocompromised host by activating the stringent response through mutation of *relA*. Precisely how VRE colonize and infect and the selective pressures that led to the outgrowth of *relA* mutants are the subjects of ongoing research.

## INTRODUCTION

Bacteria in the genus *Enterococcus* occur among the intestinal consortia of hosts that span the animal kingdom (1). These intrinsically rugged bacteria have become leading multidrug-resistant hospital pathogens. The enterococci are among the vanguard of antibiotic-resistant bacteria for at least two reasons: they evolved in the gastrointestinal (GI) tracts of insects and other invertebrates that naturally live and feed on organic matter that contains antibiotics and antibiotic-producing microorganisms (2), and they are naturally hardy and are able to withstand starvation, desiccation, and other stresses better than most other microbes. Enterococci are intrinsically resistant to many antibiotics, and they readily mutate or acquire resistance genes to others as needed. In addition to being leading multidrug-resistant hospital pathogens, they also serve as reservoirs and transmit resistance genes to other bacteria. *Enterococcus faecium* and *Enterococcus faecalis* are the enterococcal species most often associated with multidrug-resistant nosocomial infection, and about 30 years ago, both species acquired resistance to the important last-line bactericidal drug, vancomycin.

**FIG 1:**
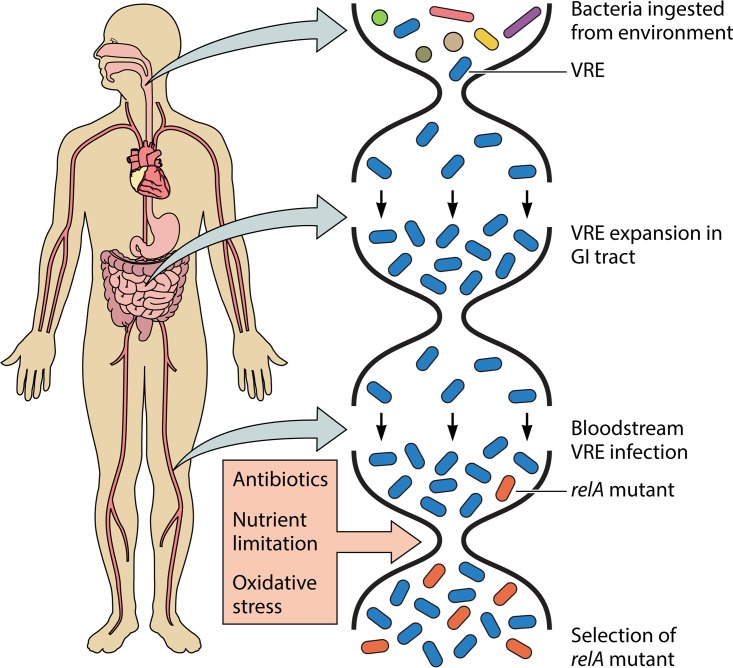
Simplified model of bacterial population dynamics during VRE colonization, infection, and selection of *relA* mutants in an immunocompromised patient. Bacteria, including VRE, are first ingested from the patient-proximal hospital environment. Transit through the upper GI tract coupled with broad-spectrum antibiotic treatment kill off nearly all other microbes, allowing VRE to grow to very high densities in the lower GI tract. A small number of bacteria from the GI tract population seed the bloodstream, where the population expands again. Once a *relA* mutant bacterium occurs in the bloodstream VRE population, it is able to grow to a high enough density to be detected, because it provides a survival advantage against antibiotic treatment, nutrient limitation, and/or oxidative stress.

Vancomycin-resistant enterococci (VRE) pose a special threat to immunocompromised patients, who often undergo antibiotic treatment or prophylaxis in hospitals. The study by Honsa and colleagues (3) describes the evolution of a vancomycin-resistant *E. faecium* strain within such a patient. How did this pediatric patient become infected with VRE? Nearly all VRE infections begin with colonization of the GI tract by bacteria that are ingested from the hospital environment (Fig. 1). Enterococci are resistant to desiccation and starvation, and they are difficult to eradicate from the hospital with disinfectants (1). From contaminated patient-proximal surfaces, including bed rails and thermometer handles, enterococci survive transit through the stomach and small intestine, and then they replicate in the large intestine where they may reach very high densities due to limited competition because of prior antibiotic treatment. Dense intestinal colonization with VRE recontaminates the patient-proximal environment, perpetuating its presence within modern hospitals (4). The risk of developing a VRE infection is very likely directly proportional to the number of drug-resistant enterococci in the GI tract; more colonization equals greater risk of infection.

The patient described in this study (3) was uniquely susceptible to vancomycin-resistant *E. faecium* colonization and infection. Only 6 weeks old, she had an immature GI tract microbiome, which was further confounded by chemotherapy to treat acute myeloid leukemia, causing impaired host defenses, accompanied by antibiotic treatment. Among the agents used was a broad-spectrum cephalosporin, to which enterococci are intrinsically resistant, and which prior work has shown limits the occurrence of competing commensal anaerobes (5–7). The VRE that colonized the patient’s GI tract most likely seeded her bloodstream, but whether that occurred through translocation through the gut wall, which often becomes inflamed during chemotherapy (8) or from contaminants that made their way to the skin and were introduced into the bloodstream through the central venous catheter is unclear.

It was recently shown that hospital-adapted, multidrug-resistant strains of *E. faecium* belong to a distinct lineage (clade A) that diverged from human commensal strains (clade B) of the species approximately 3,000 years ago (9). The lineage of hospital-adapted *E. faecium*, known as clade A1 or clonal cluster 17 (CC17), is so different from commensal strains that an argument can be made that they should be considered a different species entirely (9). The VRE strain studied by Honsa et al. (3), in fact belongs to clade A1/CC17, which is globally disseminated. Genetic factors enriched in this hospital-adapted *E. faecium* clade include the following: (i) altered cell wall polysaccharide and capsule biosynthesis genes that appear to enhance its fitness for the hospital environment (10); (ii) an accumulation of mobile elements likely due to a missing clustered regularly interspaced short palindromic repeat (CRISPR)-Cas system (9, 11); and (iii) a unique phosphotransferase system (PTS) that allows for the utilization of amino sugars, which occur on epithelial cell surfaces and mucin. This PTS has been shown to enhance the ability of *E. faecium* to colonize the antibiotic-perturbed GI tract (12), and suggests that hospital-adapted VRE strains adopt a parasitic lifestyle, feeding on host-derived secretions, such as mucin, when other dietary carbohydrates are not available.

By sequencing the genomes of 22 consecutive bloodstream VRE isolates from the same patient, Honsa and colleagues (3) were able to identify mutations that occurred during infection and growth in blood, an environment to which enterococci are not naturally adapted. A mutation in *relA* was repeatedly seen in 8 of the 22 isolates, so the authors investigated possible biological consequences of this mutation. They found that *relA* mutant strains constitutively activated the stringent response through elevated baseline levels of the alarmone (p)ppGpp. The *relA* mutant strains were not more drug resistant as defined by the ability to grow planktonically in higher levels of drug, but in biofilms, the mutant strains showed greater tolerance to lethal doses of both linezolid and daptomycin. Enigmatically, biofilms formed by the *relA* mutants were less adherent and more sparsely populated under the conditions studied.

The authors suggest that antibiotic use may have provided the selective pressure that led to the outgrowth of *relA* mutant strains. The adaptation of enterococci to growth in the bloodstream is complex, and the selective pressure or pressures may be more complex than initially thought. Daptomycin has been shown to select for *relA* mutations *in vitro* (13), and daptomycin treatment in this patient may have helped sustain the *relA* mutants at a high frequency in the population. Nevertheless, daptomycin could not have been the original selective pressure that drove the mutation to a high enough level to be detected in the first place, because the initial *relA* mutant was isolated a week prior to daptomycin therapy. Other antibiotics given to the patient preceding the appearance of the *relA* mutation were cefepime, vancomycin, meropenem, and linezolid. Of these antibiotics, vancomycin is known to have the ability to induce the stringent response (14); however, this strain was already highly vancomycin resistant and was likely inert to the effects of the drug. Use of beta-lactams and linezolid have been shown to be associated with *relA* mutations in *Staphylococcus aureus* (15, 16), but these drugs do not appear to directly induce the stringent response. Indeed, Honsa and colleagues found that linezolid exposure did not increase (p)ppGpp levels in the wild-type strain (3).

What besides antibiotic pressure may have selected for activation of the stringent response in this patient? First, nutrient limitation is well-known to activate the stringent response (17). While blood may not seem like a nutrient-limited environment, nutrient withholding is a key component of innate immunity (18), and the nutrient profile of blood differs substantially from that of the GI tract. Enterococci therefore need to alter their metabolism and physiology considerably to grow efficiently in the bloodstream. Second, exposure to reactive oxygen species during bacterial engulfment by immune cells of an immunologically normal host, and possibly with residual function in this infant receiving chemotherapy, is a potent activator of the stringent response (17). Could one or more of these mechanisms have selected for constitutive stringent response expression by the VRE isolated from the pediatric patient studied by Honsa and colleagues (3)? Despite severe neutropenia and bone marrow that was not producing functional immune cells due to chemotherapy, tissue-resident macrophages may still have been present and functioning (19). Overall, our understanding of how VRE adapt to nutrient limitation, antibiotic pressure, and oxidative stress in bloodstream infections is far from complete, and which of these may have selected for the outgrowth of bacteria with *relA* mutations in this case remains highly speculative.

Regardless of the pressure or pressures that selected for them, the *relA* mutants were unable to completely displace the wild-type strain within the bloodstream of this patient. Only 8 of the 22 isolates collected possessed the *relA* mutation, and wild-type strains were isolated throughout the course of the infection. Honsa and colleagues (3) showed that the *relA* mutants were compromised in their ability to form biofilms; this finding demonstrates how context matters when assessing whether a mutation is beneficial or not. In the face of cellular stresses, such as those described above, mutating *relA* in order to accumulate (p)ppGpp appears to be beneficial. However, in other situations, such as needing to compete for resources in order to grow and persist in the bloodstream, the same mutation is likely detrimental. For the population as a whole, it seems that retaining a mixture of wild-type and *relA* mutant bacteria might have offered the best of both worlds.

Ultimately, bloodstream infection with VRE is likely an evolutionary dead end for the bacteria. Because VRE are transmitted between patients by the fecal-oral route, bacteria growing in the bloodstream are highly unlikely to find their way into a subsequent patient. Thus, bacteria must “relearn” how to grow within the bloodstream of each new patient that they infect. Honsa and colleagues (3) show that activation of the stringent response is an advantageous adaptation in the bloodstream of an immunocompromised host, and this is the first study to document an *E. faecium* strain evolving a *relA* mutation *in vivo*. Future surveillance will determine whether this adaptation is generally encountered in enterococcal bacteremia or whether the outgrowth of these mutants was unique to the ecological conditions present in the bloodstream of this infant.
